# A Passive Microfluidic Device for Chemotaxis Studies

**DOI:** 10.3390/mi10080551

**Published:** 2019-08-20

**Authors:** Maria Laura Coluccio, Maria Antonia D’Attimo, Costanza Maria Cristiani, Patrizio Candeloro, Elvira Parrotta, Elisabetta Dattola, Francesco Guzzi, Giovanni Cuda, Ernesto Lamanna, Ennio Carbone, Ulrich Krühne, Enzo Di Fabrizio, Gerardo Perozziello

**Affiliations:** 1 Department of Experimental and Clinical Medicine, University “Magna Graecia” of Catanzaro, 88100 Catanzaro, Italy; 2 Department of Chemical and Biochemical Engineering, Technical University of Denmark, 2800 Kgs. Lyngby, Denmark; 3 Physical Science and Engineering Division, King Abdullah University of Science and Technology, Thuwal 23955-6900, Saudi Arabia

**Keywords:** chemotaxis, passive microfluidic device, diffusion, mini incubator

## Abstract

This work presents a disposable passive microfluidic system, allowing chemotaxis studies, through the generation of a concentration gradient. The device can handle liquid flows without an external supply of pressure or electric gradients, but simply using gravity force. It is able to ensure flow rates of 10 µL/h decreasing linearly with 2.5% in 24 h. The device is made of poly(methylmethacrylate) (PMMA), a biocompatible material, and it is fabricated by micro-milling and solvent assisted bonding. It is assembled into a mini incubator, designed properly for cell biology studies in passive microfluidic devices, which provides control of temperature and humidity levels, a contamination-free environment for cells with air and 5% of CO_2_. Furthermore, the mini incubator can be mounted on standard inverted optical microscopes. By using our microfluidic device integrated into the mini incubator, we are able to evaluate and follow in real-time the migration of any cell line to a chemotactic agent. The device is validated by showing cell migration at a rate of 0.36 µm/min, comparable with the rates present in scientific literature.

## 1. Introduction

The generation of chemical gradients is very widespread in the biological field because, in living organisms, the cellular environment is rich in chemical–physical signals that interact with living systems through concentration gradients [1,2]. These generate the directed motion of cells towards or away from chemical attractants (food, nutrients) or repellants (toxins). Chemical gradients play roles in a wide range of biological processes: cell development, cell differentiation, inflammation [3,4], wound healing [5] and cancer metastasis [6]. The study of these phenomena requires exposition of cells to stimuli that are quantifiable, controllable and mimic those that are present in vivo [7]. Given the biological and physiological importance of chemotaxis, understanding its mechanisms has become one of the most important goals in research about cell migration. Although, even today, there are no ideal tests for chemotaxis studies, there are numerous protocols and devices on the market that allow good correspondence [8]. Among the conventional assays, we mention: (i) The PP-Chamber, a dish filled with agar in which cells and substances to test are placed in different positions. Both diffuse in the Petri dish with a gradient, which is not stable over time [9]. (ii) The Boyden’s chamber, which contains two compartments separated by filtering membranes. The chemo-actives spread from the lower to the upper compartment through the membrane generating a gradient, while cells, loaded into the upper compartment, migrate through the membrane in response to the chemo-attractors gradient and consequently are counted at the end of the experiment [10]. (iii) The Zigmond’s chamber, consisting of two parallel channels incised in a slide and among these there is a glass ledge. Cells are seeded on a glass coverslip and the latter is then turned upside down on the channels. The gradient is formed in the thin space above the glass ledge, because the sink channel is filled with the culture medium and the source channel is filled with the biomolecule solution [11]. (iv) The Dunn’s chamber, very similar to that of Zigmond, in which the sink and source chamber have the shape of concentric rings. They are filled with the appropriate solutions before being covered with the glass coverslip where cells are seeded [12].

There are several disadvantages for such conventional techniques: For example, they do not allow the real-time quantification of cell migration at the single cell level and do not allow maintaining and control of chemical gradients, since liquids in such devices are in a static condition. These limitations have been partly overcome by microfluidic devices [13,14,15], which are nowadays widely used for several applications, studying cell biology [16,17,18] and also in studies of chemotaxis, mainly due to the high level of functional elements [19,20,21], sensor integration [22,23] and microenvironment control [23,24,25] obtainable in such devices. They are able to create dynamic environments in which the gradient can be kept constant, have a low consumption of reagents and allow quantitative evaluations of cell migration. In one of the most recent studies of chemotaxis using a microfluidic device, Ke Yang et al. developed a triple-unit gradient-generating microfluidic device. Among the main features of this device, there is the standalone generation of the concentration gradient, the docking structure for cell alignment, the possibility of simultaneously performing multiple experiments of chemotaxis on the single chip and the quantification of migration at the single cell level without time-lapse [26]. Also, recently, Jing Song et al. [27] developed a microfluidic device for the study of chemotaxis with respect to the targeting of cancer bacteria. The device consists of a central channel of bacteria at the sides extending to two cell culture chambers (normal cells on one side and tumors on the other), connected to the central channel by use of microchannels. Cells produce biochemical factors that lead to the generation of a concentration gradient in the microchannels. Once the bacteria such as *Escherichia coli* are inserted into the central channel, it is possible to observe the migration of these bacteria towards the tumor cells. However, current microfluidic systems require complicated setups and are not easy to use. Furthermore, an additional disadvantage of conventional assays for chemotaxis studies is that biologists must manually introduce various culture media daily. The here presented device is a passive automated system, which can potentially simplify protocols and standard operating procedures during chemotaxis studies. It does not need any complicated instrumentation to handle the liquids: Once the device is primed with media and samples, these are driven by gravity acceleration. It is an open system, giving access to the cell chamber facilitating sample loading and retrieval by means of a simple pipette. The experiments can be observed under a microscope integrating a mini incubator which allow temperature and humidity control. It is made in Plexiglas and fabricated by using micro-milling and solvent assisted bonding, which are low cost materials and technologies, which allow the use of the device as disposable. In addition, Plexiglas is a highly biocompatible material and ensure a good mechanical stability.

## 2. Materials and Methods

### 2.1. Materials

For the microfluidic device (MFD) fabrication, the layers of poly(methylmethacrylate) (PMMA; 3 mm thick) were purchased from Röhm Italia Srl (Garbagnate Milanese, Italy). The ethanol, used for the bonding process, was purchased from Sigma-Aldrich (St. Louis, MO, USA). For the device functionalization, the polyethylene glycol (PEG; molecular weight of 4000 g/mol) was purchased from Sigma-Aldrich. For the experiments with cells, the Rosewell Park Memorial Institute 1640 medium (RPMI 1640), used as culture medium, was purchased from Sigma-Aldrich; fetal bovine serum (FBS) used mixed fifty–fifty with RPMI as enriched medium was purchased from Sigma-Aldrich, which then acted as a chemoattractant, as the cells, cultured in the RPMI only, tended to move towards nutrients. The cells used were Jurkat, namely acute T cell leukemia lymphoblasts, purchased from ATCC (Sesto San Giovanni, Italy). The cell concentration used was 1 million/mL.

### 2.2. Working Principle

**Figure 1:**
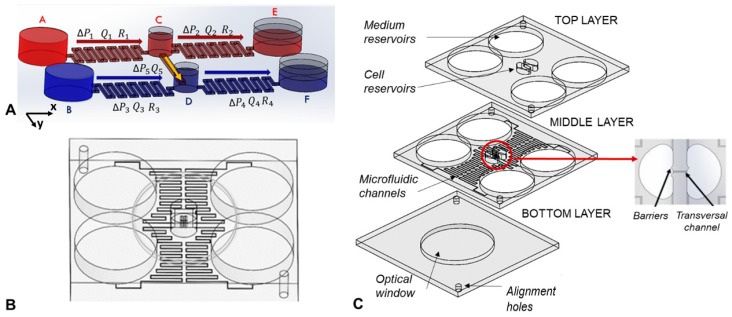
(**A**) Device scheme (not in scale); (**B**) CAD drawing of the microfluidic device; (**C**) exploded view of the device. All the CAD files can be found in Supplementary Materials.

The scheme of the device layout is shown in Figure 1A. It was composed of two symmetric parts connected by a transversal channel. A microfluidic channel connected the source reservoirs (A and B) and the central reservoirs (C and D) and a second microfluidic channel which had the same fluidic resistance (R1 = R2 and R3 = R4) connected the central reservoirs and the drain reservoirs (E and F). In addition, a transversal channel connected the two central reservoirs. The functionality of the device allowed creation of a constant concentration gradient in the transversal channel. This was obtained by flowing two liquids at different concentrations from the source reservoir to the drain reservoir in each part of the device. The same hydrostatic pressure (P_C_ = P_D_) and same flow in inlet and outlet in the central reservoirs (Q_1_ = Q_2_ and Q_3_ = Q_4_) was kept to avoid flows along the transversal channel (ΔP_5_ = P_C_ − P_D_ = 0, Q_5_ = 0). The liquids were driven in the microchannels by gravity. Exploiting the symmetry of the device and setting the proper volume of liquids in the reservoirs, hydrodynamic flows along the x-direction and only diffusion of molecules in y-direction (through the transversal channel) were obtained. 

### 2.3. Device Fabrication

The device layout is shown in Figure 1A,B. It was composed of three layers of PMMA (80 cm long, 72 cm wide and 3 mm high) which were machined by micro-milling (Mini-Mill/Gx from MiniTech machinery corporation, Peachtree Corners, GA, USA) in order to reproduce, on the different layers, the reservoirs, the microfluidic channels, and two alignment holes. In particular, the top layer integrated part of the source and drain reservoirs used for the cell media (3 cm in diameter) and central reservoirs used for the cell samples (which had a half-circular shape of 1 cm in diameter) and two alignment holes (3 mm in diameter). The middle layer integrated part of the media reservoirs and sample reservoirs, four microfluidic channels (2 cm long, 185 μm wide and 140 μm high) connecting the media reservoirs with the sample reservoirs, and a transversal micro channel (2 mm long, 185 μm wide and 140 μm high) connecting the sample reservoirs in which the concentration gradient would be formed. At the inlet and outlet of the transversal microchannel, a barrier was integrated to prevent the cells from being washed away by the flows. The bottom layer integrated the optical window (40 mm in diameter) and two alignment holes (3 mm in diameter). The three PMMA layers were bonded together by solvent assisted bonding. During this process, the three layers of PMMA were placed in ethanol for 1 h and 20 min, were then aligned by using the alignment holes and pressed together at 1.5 kN at a temperature of 45 °C for 1 h in a heat press (PO Weber^®^, Remshalden, Germany) [28]. Before carrying out the experiments, the device was treated with a 10% aqueous solution of PEG (polyethylene glycol) dissolved at 30 °C, to improve its wettability. The solution is introduced into the channels by a syringe with suction cup and was then evaporated with nitrogen flow.

### 2.4. Experimental Set-Up

**Figure 2:**
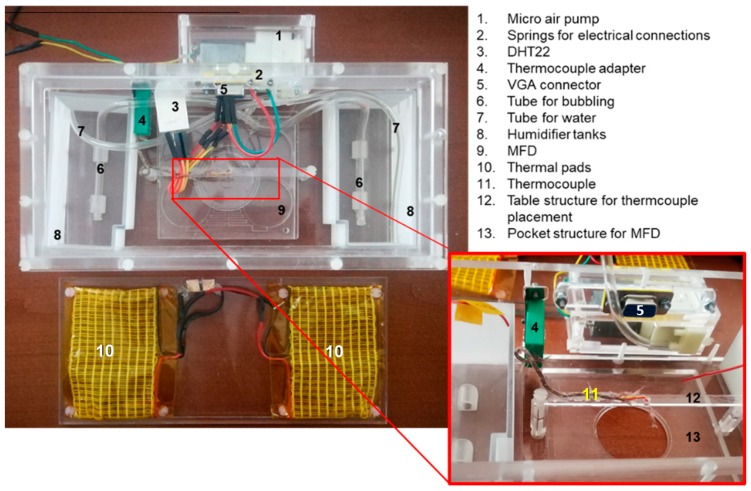
Experimental set-up used to characterize and validate the microfluidic device: mini-incubator and microfluidic device (MFD).

For the experimental validation, a miniaturized incubation chamber, designed such that it can integrate a passive microfluidic device, was fabricated and coupled to an inverted microscope stage (NIKON Eclipse Ti, Firenze, Italy). This allowed (i) to control temperature and humidity levels, preventing evaporation during cell cultivation, (ii) to assure a contamination-free environment for cells containing air and 5% of CO_2_ and (iii) to monitor flows and cells over time. The mini incubator (210 × 102 × 59 mm) was made-up in PMMA by means of milling and 3D printing. The MFD could be positioned at the centre of the incubator in correspondence of an optical window, allowing the optical screening of the MFD by means also of high magnification objectives (up to 60×) with a working distance down to 1.8 mm. Figure 2 shows the mini-incubator/MFD setup and the automated temperature control unit based on a programmable open source microcontroller (Arduino Uno). The miniaturized incubation chamber also integrated a 5% CO_2_ source, a humidification system, a thermocouple placed near the microfluidic device, a thermal pad heated by an external electrical power source, an environmental temperature and a humidity sensor.

### 2.5. Simulation of the Concentration Gradient

A computational fluid dynamics (CFD) simulation allowed the diffusion of the dye inside the transversal channel to be simulated and followed to compare results with the experimentally determined gradient. The simulation was carried out on the transversal channel considering the diffusion of the food dye and of a chemotactic factor (such as interleukin 8; IL-8).

What varied in the two simulations was the diffusion coefficient, which for IL-8 was equal to 2.5 × 10^−10^ m^2^/s [29], while for food dye (mainly complex carbohydrates) was equal to 1 × 10^−12^ m^2^/s, similar to that of starch [30]. Starch is an organic compound of the carbohydrate class, characterized by a large number of polymerized glucose units, it is larger than the other components and we can therefore consider the behaviour of the dye similar to that of starch.

### 2.6. Device Characterization—Concentration Gradient

Food-grade gel dyes were used to visualize and measure experimentally the diffusion in the transversal channel of the device. The dye was dissolved in water (ratio 1:10) heated to 45 °C, stirred until a homogeneous colour and the absence of lumps was obtained. The diluted solution was then loaded in the source reservoirs of the device and diffusion was observed under the optical microscope. The observation was performed for 24 h with time-lapse images taken at 10-min intervals at 21 different locations. By observing the colour change in the channels over time, it was possible to qualitatively monitor the direction of the liquid flow inside the device. In addition, the concentration gradient of the dye along the channel, as a function of the time, was determined with the help of ImageJ, bundled with Java 6, software (National Institutes of Health (NIH), Bethesda, MD, USA), correlating the concentration to different pixel intensities along the channel of images recorded in time-lapse.

### 2.7. Device Characterization—Chemotaxis Experiments

The device was validated by investigating the chemotaxis of Jurkat cells, attracted by a medium containing FBS (used as chemoattractant). For such a purpose, the device was sterilized by autoclaving. One part of the device was primed with a culture medium (RPMI 1640) and the other part with the enriched medium (RPMI 1640 with a 50% concentration of FBS). Cells were loaded in the central reservoir. The cellular movement towards the nutrient source could be observed. The used cell concentration was 1 million/mL.

The protocol used to perform these experiments with cells was the following:

1. The channels were primed with the culture medium.
2. The volumes accumulated in the reservoirs were emptied in the priming phase.
3. The drain (E and F in Figure 1A) was filled with the appropriate volumes of culture media (1320.4 μL).
4. The source (A and B in Figure 1A) was filled with the appropriate volumes: one (A) with the enriched medium (3750 μL), the other (B) with the culture medium (3961 μL).
5. The central reservoirs were filled: One with culture medium, the other with cells. For this step a multi-channel pipette was used, to introduce simultaneously the same volumes inside the reservoirs, in order to avoid cell flows inside the transverse channel.

## 3. Results and Discussion

**Figure 3:**
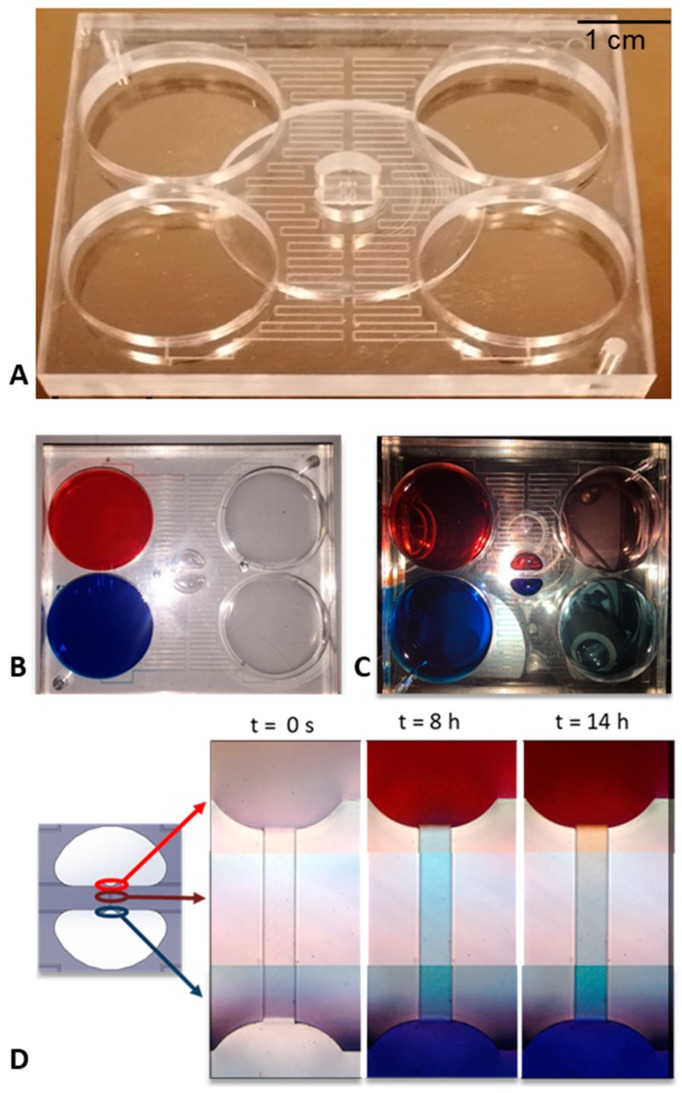
Isometric view of the microfluidic device (**A**); the device in the loading phase of the dyes appears as in the figure (**B**), while at the end of the experiment there is a fairly uniform concentration within the central reservoirs and in the drain ones, maintaining, however, two flows at different separate concentrations (**C**). We also report the time-lapse acquisitions of the channel at the most significant time instants (**D**).

After fabricating the layers composing the device by micro-milling and having carried out the solvent assisted bonding, the final device appeared as in Figure 3A.

### 3.1. Simulation Results

**Figure 4:**
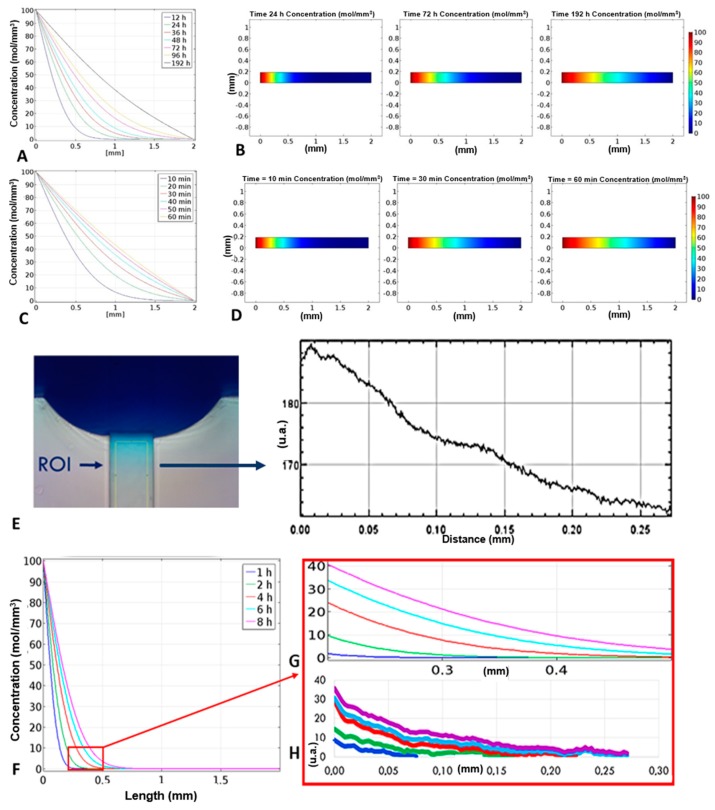
(**A**,**B**) Simulation of the food dye concentration gradient: the concentration gradient of the dye within the transversal channel is linear only after 192 h, due to the low value of the diffusion constant; (**C**,**D**) simulation of the interleukin-8 (IL-8) concentration gradient: unlike the food dye, the concentration gradient of the interleukins becomes linear after almost 60 min; (**E**) the concentration values were calculated according to the pixel intensity of images taken in time-lapse along the transversal channel and are therefore expressed in arbitrary units, we report the pixel intensity as a function of the distance 8 h after having filled the device with the blue dye; (**F**) gradients of concentration of the food dye as a function of the channel position at different times; (**G**) simulated trend of the concentration gradient within the transverse channel, values expressed in mol/m^3^; (**H**) real trend of the concentration gradient.

The simulations reflect the expected theoretical behaviour of the two species: as we know, in fact, the diffusion time is proportional to the size of the molecule that spreads. As we can see in Figure 4A,B, after 12 h a gradient could be observed in the first 0.5 mm of the transversal channel and a linear gradient throughout the entire length of the channel could be observed after almost 200 h. This long time was due to the value of the diffusion constant chosen equal to 1 × 10^−12^ m^2^/s, according to the ingredients of the food dye (mainly complex carbohydrates). Usually diffusion velocities of small proteins, sugars, or other biomolecules used in chemotaxis studies are 100 times higher than the one considered and this means that a linear gradient is formed in few hours, as it can be seen in Figure 4C,D.

### 3.2. Concentration Gradient Characterization

The characterization of the device was firstly achieved by placing the device under an optical microscope, (NIKON Eclipse Ti) loading the two source reservoirs of the device with two solutions containing two different dyes (red and blue) and looking how these were driven in the channels at different times. As it can be seen from Figure 3D, after 8 h a net flow from the blue reservoir to the red one was observed, which is why the channel appears blue. These net flows take place due to several reasons. First, even small inclinations of the stage can cause this phenomenon. In addition, the real fluidic resistances of the microfluidic channel, due to technological limits of the micro-milling machine could be slightly different from the theoretical ones, meaning that the flow rates between the source and drain reservoirs were different too. In this situation, in the first hours a net flow was present in the transversal channel, which did not allow a concentration gradient to be obtained along it. Then, the system rebalanced itself and, after 14 h, a gradient could be observed in the transversal channel. It has to be noticed that, in these experiments, the gradient formation was slow because of the low diffusion coefficient of the used samples.

The gradient formation, calculated as difference between concentration in a point along the transversal channel and the beginning of the channel, was optimized modulating the volumes of liquids in the reservoirs A and B loaded with dye diluted in water and water, respectively. For each couple of volumes in A and B, the concentration gradient of the dye along the channel, as a function of time, was determined capturing the images along the channel in time-lapse and, with the help of the ImageJ software, correlating the concentration to the different pixel intensities. Then, the CFD simulation was compared with the experimental gradient. The simulated and experimental results were in alignment (Figure 4G,H), the dye diffused slowly through the transversal-channel, due to its low diffusion coefficient, equal to 1 × 10^−12^ m^2^/s, chosen according to the ingredients of the food dye (mainly complex carbohydrates). Fits of the data representing the concentration gradient formation as function of time are reported in Supplementary Materials (Figure S1).

**Figure 5:**
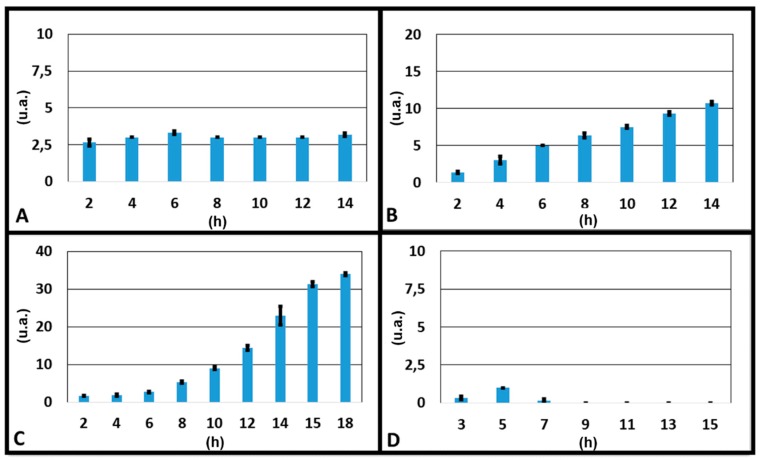
Graphs of the gradients as a function of time obtained during the experiments with dyes in which: (**A**) 3000 µL of water + dye and 3500 µL of only water were loaded respectively in the two source reservoirs; (**B**) 3500 µL of water + dye and 3500 µL of only water were loaded respectively in the two source reservoirs; (**C**) 3750 µL of water + dye and 3500 µL of only water were loaded respectively in the two source reservoirs; (**D**) 3961 µL of water + dye and 3500 µL of only water were loaded respectively in the two source reservoirs.

To understand the better condition for an optimal gradient along the transversal channel, we report the results of four experiments in which volumes of the two reservoirs A and B were varied (3000 and 3500 μL, 3500 and 3500 μL, 3750 and 3500 μL and 3961 and 3500 μL respectively). The colour intensity at the inlet and outlet of the transversal channel for each experiment was measured, and the correspondent concentration gradient was calculated as the difference between the outlet and inlet values, at different times (Figure 5). It could be seen that the optimal gradient was not reached when the volumes of liquid were the same in the two source reservoirs (3500 and 3500 μL), which were the expected results, since the device was symmetric. This confirmed what was observed above: the gradient formation was affected by some factors, such as a difference in resistance between the channels due to limits of the technologies used for fabricating the device and from a lack in planarity of the microscope stage where the device was mounted during experiments. Nevertheless, we had different results when we put a different volume of liquid in only one reservoir. The optimal result was reached when 3750 μL in the source reservoir A, 3500 μL in the source reservoir B and 1320 μL in the drain reservoirs (E and F) were loaded.

### 3.3. Chemotaxis Results

**Figure 6:**
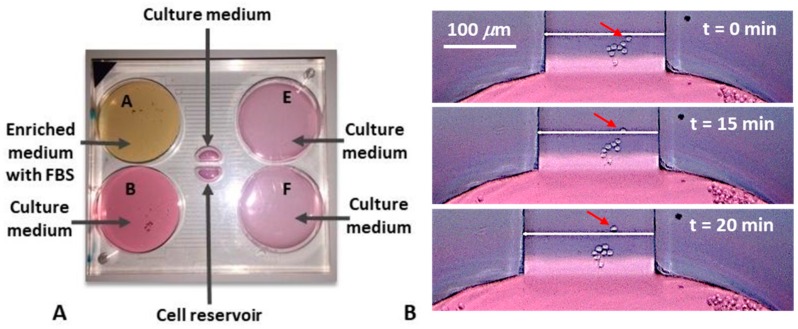
Cells media within the device (**A**); chemotaxis of a single cell (**B**). Cell movements could be observed after about 175 min. The average speed of cell movement was about 0.36 μm/min.

After inserting the media with and without chemotactic factors and the cells inside the device and establishing the nutrient concentration gradient inside the transverse channel, we could observe the cellular movement towards the nutrient source. In the Figure 6B, it is possible to observe the chemotaxis of a single cell, which occurred in about two and a half hours. Of notice, the other cells did not change position, which means that there were no streams. Furthermore, the average speed at which the cells moved was measured and it was 0.36 μm/min, well in alignment with the results found in the scientific literature [31].

Chemotaxis experiments were performed also with both source reservoirs containing the same medium to validate the precedent results, and chemotaxis was not observed. Chemotaxis, in fact, occurred when cells moved in search of nutrients (e.g., glucose) towards the maximum concentrations of molecules of their interest. If there was not a gradient concentration, there was not the drive force to stimulate cells’ movements, as it happened in the case in which all the reservoirs contained the same media.

## 4. Conclusions

In conclusion, we showed a passive microfluidic device in which it was possible to perform chemotaxis experiments. The realized geometries have allowed the obtainment and visualization, by means of colour dyes, flows and diffusion inside the channels. The sizing of the microchannels allowed the obtainment of almost constant flow rates of the order of a few tens of microliters per hour (~10 μL/h) within the microchannels, decreasing by 2.5% in 24 h, which did not affect cell adhesion to the substrate. Furthermore, the calibration of the volumes in the reservoirs allowed the generation of a concentration gradient inside the transversal channel and the subsequent visualization of the chemotaxis phenomenon.

## Supplementary Materials

The following are available online at https://www.mdpi.com/2072-666X/10/8/551/s1, Figure S1: Concentration gradient formation in time-lapse and fitting curves, CAD file S1: Microfluidic device and Optical window 1, CAD file S2: Microfluidic device and Optical window 2, CAD file S3: Mini incubator 1_hidden components, CAD file S4: Mini incubator 1, CAD file S5: Mini incubator 2_hidden components, CAD file S6: Mini incubator 2.
