# Supplementary material for: A Passive Microfluidic Device for Chemotaxis Studies
Source: Micromachines (Basel). 2019 Aug 20;10(8):551. doi: 10.3390/mi10080551 (PMC6722731; doi:10.3390/mi10080551)
Supplement: Supplementary file 1 [file micromachines-10-00551-s001.zip › Supplementary materials/micromachines-558919-supplementary materials .docx]

Supplementary Materials

A Passive Microfluidic Device for Chemotaxis Studies

Maria Laura Coluccio, Maria Antonia D’Attimo, Costanza Maria Cristiani, Patrizio Candeloro, Elvira Parrotta, Elisabetta Dattola, Francesco Guzzi, Giovanni Cuda, Ernesto Lamanna, Ennio Carbone, Ulrich Krühne, Enzo Di Fabrizio and Gerardo Perozziello

Fits of the data representing the concentration/flow behaviour (or concentration gradient formation) as function of time.

The gradient formation, calculated as difference between concentration in a point along the transversal channel and the beginning of the channel, was followed, for different couples of volumes in reservoirs A and B, as a function of time, capturing the images along the channel in time lapse and, with the help of the ImageJ software, correlating the concentration to the different pixel intensities.

The data can fit with a sigmoidal curve of equation:

| $y=\frac{34}{1+{1,7}^{-\left( t-12,5 \right)}}$ | (1) |
| --- | --- |

Theoretical and experimental data are reported in the following graph:

**Figure S1.** Theoretical (red points) and experimental (blue points) data of the concentration gradient formation in time-lapse and fitting curves.

The first part of the graph is dominated by net flows, while once the flows are stabilised, the gradient formation is faster and at a certain point saturates remaining constant.

© 2019 by the authors. Submitted for possible open access publication under the terms and conditions of the Creative Commons Attribution (CC BY) license (http://creativecommons.org/licenses/by/4.0/).
